# Impact of Footwear and Foot Deformities in patients with Parkinson's disease: A Case-Series Study

**DOI:** 10.7150/ijms.50967

**Published:** 2021-01-01

**Authors:** Eloy Novo-Trillo, Daniel López-López, Carmen de Labra, Marta Elena Losa-Iglesias, Ricardo Becerro-de-Bengoa-Vallejo, César Calvo-Lobo, Carlos Romero-Morales, Marta San-Antolín-Gil

**Affiliations:** 1 Research, Health and Podiatry Group. Department of Health Sciences. Faculty of Nursing and Podiatry. Universidade da Coruña, Ferrol, Spain.; 2 NEUROcom, School of Health Sciences University of A Coruna, and Agrupación estratégica CICA-INIBIC - UdC, A Coruna, Spain.; 3 Faculty of Health Sciences. Universidad Rey Juan Carlos, Spain.; 4 Facultad de Enfermería, Fisioterapia y Podología. Universidad Complutense de Madrid, Spain.; 5 Faculty of Sport Sciences, Universidad Europea de Madrid, Villaviciosa de Odón, Madrid, Spain.; 6 Department of Psychology, Universidad Europea de Madrid, Villaviciosa de Odón, Madrid, Spain.

**Keywords:** Parkinson's disease, footwear, foot deformities

## Abstract

**Background:** Parkinson's disease (PD) is a common and complex neurological problem. Gait abnormalities are frequent in PD patients, and this increases the risk of falls. However, little is known about foot deformities and footwear in this vulnerable population. Here we investigate whether patients with PD use an appropriate shoe size and know if they have foot deformities or alterations.

**Methodology:** A study of a series of observational descriptive cases in a convenience sample (n = 53 patients) diagnosed with Parkinson's disease. One trained investigator evaluated foot and ankle health. The footwear and foot measurements were obtained using a Brannock device.

**Results:** The podiatric examination and footwear examination detected a high presence of podiatric pathologies and inappropriate footwear. This has a negative impact on the quality of life of these patients.

**Conclusions:** This research detected an elevated number of people with foot deformities or alterations. Moreover, a high proportion of participants with PD wear inadequate footwear (in length, width, or both).

## Introduction

Parkinson's disease (PD) is the most frequent movement disorder and represents, after Alzheimer's disease, the principal neurodegenerative problem related to the central nervous system 1. PD affects 1% of people > 60 years old and has a prevalence of 3% among those > 80 years old 2. The PD population is expected to increase by more than 50% by 2030 3, costing the European healthcare system an estimated 9000 million 4.

The risk factor best associated with the beginning of PD is age. Genetics plays a role in those where the disease manifests before 50 years old 5,6. Tobacco smoking is associated with a lower risk of PD, even though the mechanism of this relationship remains unknown 7,8. Other risk factors (environmental exposures, melanoma, diabetes, hypertension, alcohol, high milk and dairy consumption, and obesity) and protective (caffeine consumption, vitamin E, a non-steroidal anti-inflammatory drug, urate, physical activity of intensity moderate, calcium channel blocker, and statins) present contradictory results 9,10.

Today, PD is understood as a multifactorial condition with idiopathic etiopathogenesis 11. Its onset is insidious, which makes it complicated to diagnose in the initial stages, starting unilaterally 12. The crucial pathological feature of PD is the decrease of dopamine cells in the substancia nigra pars compacta; another hallmark is Lewy pathology, the protein α-synuclein accumulates abnormally inside in cell body and degenerates neurons of the nervous system 13. These results in a movement disorder characterized by the following motor signs: tremor, bradykinesia, muscular rigidity, and postural instability 14.

The relationship between foot symptoms and PD include alterations in the cycle of gait, characterized by a decrease in stride length 15. PD patients manifest significant differences in heel contact and a lowered foot uprising during the swing phase 16; hence they have an elevated risk of falling 17. This is exacerbated among people who have freezing of gait (FOG) 18.

Falls represent an important problem. Between 60 and 70% of persons with PD fall each year, with social and health consequences, including reduced quality of life and increased hospitalization, fractures, health spending, and mortality 19.

Postural instability responds poorly to drug therapy, so it is essential to investigate new action strategies. Recent research shows that plantar foot stimulation has a positive effect on the step and stride length and has a positive effect on walking stability 20. A type of mechanical foot and ankle device has been shown to improve walking and decrease the recurrence of FOG 21. The range of motion (ROM) of the ankle is altered, so performing therapeutic actions on this joint will help improve the cycle of gait 22.

Foot dystonia is frequent and might appear at the beginning of PD as an initial manifestation of the disease, inducing an abnormal posture with inversion of the foot and extension of the Hallux 23. This negatively affects the quality of life, producing pain and making it difficult to wear footwear 24. Although botulinum toxin is routinely used as a therapeutic, more studies are needed to support the notion that this is an effective treatment 25,26.

Foot problems affect posture and can induce falls among the elderly 27,28. We found only one study that addresses this topic. The authors used of a 22-question survey and found that half suffer from foot problems, and a third find it difficult to wear footwear 29. Also, no studies have analyzed footwear, which can alter gait patterns in people and is recognized as a modifiable risk factor in falls prevention 30,31. Because of this, laser shoes have been designed to alleviate FOG, offering a promising intervention in the future 32.

Here we analyze whether patients with PD use an appropriate shoe size and are knowledgeable about whether they have foot deformities or alterations. Our hypothesis is that patients with PD present foot disorders and wear inadequate footwear.

## Materials and Methods

### Design

Study of a series of observational descriptive cases 33, describing the state of health of the foot and its relationships with footwear, to monitor their health in patients with PD.

### Sample

Fifty-three subjects diagnosed with PD participated in the study between 28 January 2020 and 3 March 2020. Participants were recruited by convenience sampling.

Inclusion criteria consisted: signed informed consent, individuals diagnosed with PD by their specialist physician and not having compromised balance. Individuals that are in stage I and II, according to Hoehn and Yahr stage. The exclusion criteria include having a significant cognitive impairment that prevents them from consenting to participate in the study and understanding the guidelines of the test indicated, or having a surgical history of the lower limb or a recent fracture that prevents the person from performing the study.

### Procedure

One trained investigator picked up the measurements, following the protocol of previous studies in other vulnerable populations, such as people with Alzheimer's disease and rheumatoid arthritis 34,35.

Initial measures of the participants include: 1) demographic variables (age and gender); 2) anthropometric variables (height in centimeters, weight in kilograms, with the patient in orthostatic position and barefoot); and 3) variable body mass index (BMI) calculated from the height in meters and weight in kilograms applying Quetelet's equation (BMI = weight / height^2^) 36.

A physical examination of the foot and ankle was carried out to determine the presence of podiatric pathologies. The general health of the foot was evaluated, observing the state of the skin, and giving attention to the presence of hyperkeratosis or nail diseases 37.

ROM of the first metatarsophalangeal joint (one MTP) was assessed using a goniometer 38. Functional Hallux Limitus was considered present when one MTP did not reach 65º in extent. Hallux Rigidus was considered present when there is no movement in the extension of one MTP or this plus less than 10º. In parallel, the ROM of the ankle joint (AJ) to dorsiflexion was assessed using the Silfverskiöld test 39. Equinus deformity was considered present when AJ did not possess 10º at dorsiflexion.

The presence of Hallux Abductus Valgus (HAV) and digital deformities were tested with the patient in a bipedal position. The Manchester Scale 40 was used to catalog HAV deformity. This clinical stopover determines four levels of HAV: none, mild, moderate, and severe. It shows excellent interobserver reliability and, in contrast to radiographs, shows high interrater reliability (intraclass interrelation coefficient bigger than 0.96) 41.

We measured both the foot and the shoe size using a Brannock device, a validated measuring instrument (MedicalTtrain; Madrid, Spain). The foot exploration consisted of assessing the longitude and broadness of the foot using the validated Brannock device 42. This instrument has been shown to be safe, with an intraclass interrelation coefficient of 0.96-0.99 43. Every patient remained in an orthostatic position, barefooted, and with their weight evenly distributed, with the objective of ensuring that the tissues of the foot have extended and feet acquired maximum size. The investigator put the foot of the patient in the device, with the heel positioned in the heel cup. Then, a moveable arch length pointer is placed in the first metatarsal head. The highest number is then used in the moveable width bar. An identical protocol was used to evaluate the other foot, as well as to analyze shoe size.

The sample size was calculated with a statistic calculator designed by Unidad de Epidemiología Clínica y Bioestadística 44. The calculations took into account the number of individuals with PD in the autonomous community of Galicia, which has a total population of 2,699,499 45, with 4.91% of the population having PD 46.

To estimate a predominance of pathologies in the ankle-foot complex of 50% (p = 0.5) with a confidence level of 95% (α = 0.05), the precision of ± 14% and assuming an information loss of 8%, it is necessary to evaluate 53 people.

### Statistical Analysis

Statistical analyses were performed using SPSS 24.0 (IBM Corp, Armonk, NY, USA). P-values < 0.05 were considered statistically significant.

Sociodemographic and anthropometric variables were inquired for normal distribution using the Kolmogorov-Smirnov test, and data were considered normally distributed if *p >* 0.05. An independent Student t-test was carried out to find if differences between man and woman with PD were statistically significant in age, height, weight, and BMI.

Categorical variables appear as total values and percentages, while the quantitative variables are represented as mean, standard deviation, and maximum and minimum values. The Chi-squared (χ^2^) test was used to collate the categorical variables of foot and shoe measurements.

### Ethical Considerations

This investigation was authorized by the Research and Ethics Committee of the University of Coruña, file number 2019-0018. This study followed the principles of the Declaration of Helsinki, and the Oviedo Convention.

Each patient was given a document on the characteristic of the study, and they had the opportunity to clarify any doubts with the researcher. Also, all participants gave their informed consent, participating freely and voluntarily in the study.

## Results

Demographic and anthropometric variables show a normal distribution (*p* > 0.05). Fifty-three patients finished every phase of the investigation process, 36 of whom were male (67.9%) and 17 female (32.1%), with a mean (± SD) age of 68.15 ± 9.73 years (range, 42 to 89 years old).

We observed statistically significant differences (*p* < 0.05) in age, body weight, and stature, but no significant differences in BMI, in which overweight is observed (mean = 27.85 ± 3.63). The initial measurements of the participants are given in **Table 1.**

The podiatric examination detected that 88.68% (n = 47) of the participants present some type of podiatric pathology. The physical examination of the skin revealed that 71.7% (n = 38) had nail disorders or hyperkeratosis; the physical examination of the joints showed that 56.6% (n = 30) had hallux limitus functional, 24.5% (n = 13) had hallux rigidus, 71.7% (n = 38) had ankle equinus, and 45.3% (n = 24) had hallux abductus valgus and deformed toes.

After metering foot and footwear size, we determine that 75.5% (n = 40) of participants for the right foot and 60.4% (n = 32) of participants for the left foot use inadequate footwear, either in length, width, or both. As shown in **Table 2**, on the right and left feet, 41.5% (n = 22) and 26.4% (n = 14) wear appropriate footwear in length but not in width. Only 24.5% (n = 13) (on the right foot) and 39.6% (n = 21) (on the left foot) of participants used properly sized footwear.

## Discussion

The results of this research detected that participants have a high presence of foot deformities and use inappropriate footwear.

Our results are in line with the findings of previous podiatry studies in other vulnerable populations relating to footwear and foot health, by means of podiatric examination. The results are in line with the study by Bowen et al., which reported that 53% of 218 respondents with PD have foot problems. Also, Son et al. observed a decrease in the ROM of the ankle joint when analyzing the kinematics of gait; this is consistent with the results of our study, in which 71.7% have equinus ankle.

Various authors 47-49 state that the use of incorrectly sized footwear in length and width measurements is significantly associated with poor foot health, decreased quality of life, and increased pain, foot deformities, dermatological alterations, and falls. They also indicated that a large proportion of the population, around 63-72%, is wearing improperly sized footwear (in length and width measurements) 50. These findings are consistent with our results, where 75.5% (right foot) and 60.4% (left foot) of participants use inappropriate footwear, and a high proportion has a foot deformity.

The results are consistent with other similar studies in aging populations without Parkinson's. For example, Tovaruela et al. state that 84.93% of a total of 166 patients with rheumatoid arthritis have foot problems, and only 38.5% are using appropriate footwear. Also, López et al. 51, in an aged sample with 100 participants, reported that 88% suffer from foot problems, and 83% use inappropriate footwear. Finally, Harrison et al. 52 reported in 100 diabetic patients that only 20% use appropriate footwear, according to measurement length and width. Furthermore, several studies have reported an increase in podiatric pathologies with aging 53,54. In our study, the podiatric examination detected that 88.68% of the participants present some type of podiatric pathology.

Approximately 70% of falls have intrinsic causes, such as turning and improper stepping, aggravated by a low sensory innervation of the foot. It is known that the presence of podiatric pathologies increases the risk of falls 55.

The research has some limitations. First, the sample size is small; it would be beneficial to include participants all over Spain that strengthen the results of the study and might recognize more variables involved. Second, despite having performed a sample size calculation, it would be advantageous to have carried out a previous pilot study, which might have improved the quality of the study. Third, only one investigator analyzed the variables of participants; future investigations would involve a minimum of two investigators, one for podiatric examination and another for footwear exploration. Finally, data contrast with a control group without PD would strengthen the data. The absence of information about demographics characteristics and podiatric history should be taken into account.

Thus, podiatrists have an important role among this vulnerable population, which includes developing prevention programs, podiatry, and footwear education. All this will have an assertive impact on foot health and welfare in this vulnerable population. Additionally, shoe companies must dispose of an appropriately wide selection of footwear that allows accommodating the real morphology of the foot, especially in the transverse arch. We propose that a wider range of widths for each shoe should be available, so that it allows accommodating feet with wider dimensions.

This study provides novel data on the presence of foot deformities and footwear in people with PD. Further studies are needed for diverse strategies and interventions that enhance the general health and foot health in people with PD.

## Conclusions

This research detected an elevated number of people with foot deformities or alterations. Moreover, a high presence of participants with PD wears inadequate footwear in length, width, or both.

## Figures and Tables

**Table 1:** Variables sociodemographic and anthropometric of the sample with PD

	Total group mean ± SD (n=53)	Male mean ± SD (n=36)	Female mean ± SD (n=17)	*p*-value*
Age (years)	68.15± 9.73 (42-89)	69.97 ± 9.25 (48-89)	64.29 ± 9.99 (42-89)	0.047
Weight (years)	75.87 ± 10.92 (52-96)	78.56 ± 9.48 (61-95)	70.18 ± 11.84 (52-96)	0.008
Height (m)	165.06 ± 8.49 (150-183)	168.78 ± 7.34 (155-183)	157.76 ± 5.36 (150-169)	0.000
BMI (kg/m^2^)	27.85 ± 3.63 (22.51-39.45)	27.57 ± 2.75 (22.84-34.89)	28.43 ± 5.07 (22.51-39.45)	0.518

Abbreviations: BMI, body mass index; SD, standard deviation; Mean ± SD, range (minimum - maximum). In all the analyses* p* < 0.05 (with a 95% confidence interval) was considered statistically significant. *Students' *t* test for independent samples was used for the analysis data.

**Table 2:** Foot and shoe measurements

Standing position	Excessive shoe width	Correct shoe width	Insufficient shoe width	Total	*p*-value*
***Right foot***					0.025
Shoe size too big	3	9	3	15	
Correct shoe size	1	13	22	36	
Shoe size too small	0	0	2	2	
**Total**	4	22	27	53	
***Left foot***					0.137
Shoe size too big	2	10	3	15	
Correct shoe size	2	21	14	36	
Shoe size too small	0	0	2	2	
**Total**	3	31	19	53	

Note: In all the analyses* p* < 0.05 (with a 95% confidence interval) was considered statistically significant. *Chi-squared test was used.

## References

[1] Kalia LV, Lang AE (2015). Parkinson's disease. Lancet.

[2] Benito Leon J (2018). Epidemiology of Parkinson's disease in Spain and its contextualisation in the world. Rev Neurol.

[3] Dorsey ER, Constantinescu R, Thompson JP (2007). Projected number of people with Parkinson disease in the most populous nations, 2005 through 2030. Neurology.

[4] Maresova P, Klimova B, Novotny M, Kuca K (2016). Alzheimer's and Parkinson's Diseases: Expected Economic Impact on Europe-A Call for a Uniform European Strategy. J Alzheimers Dis.

[5] Schapira AH, Jenner P (2011). Etiology and pathogenesis of Parkinson's disease. Mov Disord.

[6] Xie F, Gao X, Yang W (2019). Advances in the Research of Risk Factors and Prodromal Biomarkers of Parkinson's Disease. ACS Chem Neurosci.

[7] Delamarre A, Meissner WG (2017). Epidemiology, environmental risk factors and genetics of Parkinson's disease. Presse Med.

[8] Searles Nielsen S, Gallagher LG, Lundin JI (2012). Environmental tobacco smoke and Parkinson's disease. Mov Disord.

[9] Ascherio A, Schwarzschild MA (2016). The epidemiology of Parkinson's disease: risk factors and prevention. Lancet Neurol.

[10] Gazewood JD, Richards DR, Clebak K (2013). Parkinson disease: an update. Am Fam Physician.

[11] Obeso JA, Stamelou M, Goetz CG (2017). Past, present, and future of Parkinson's disease: A special essay on the 200th Anniversary of the Shaking Palsy. Mov Disord.

[12] Tolosa E, Wenning G, Poewe W (2006). The diagnosis of Parkinson's disease. Lancet Neurol.

[13] Dickson DW (2018). Neuropathology of Parkinson disease. Parkinsonism Relat Disord.

[14] Sveinbjornsdottir S (2016). The clinical symptoms of Parkinson's disease. J Neurochem.

[15] Creaby MW, Cole MH (2018). Gait characteristics and falls in Parkinson's disease: A systematic review and meta-analysis. Parkinsonism Relat Disord.

[16] Kimmeskamp S, Hennig EM (2001). Heel to toe motion characteristics in Parkinson patients during free walking. Clin Biomech.

[17] Sofuwa O, Nieuwboer A, Desloovere K, Willems AM, Chavret F, Jonkers I (2005). Quantitative gait analysis in Parkinson's disease: comparison with a healthy control group. Arch Phys Med Rehabil.

[18] Son M, Cheon SM, Youm C, Kim Y, Kim JW (2018). Impacts of freezing of gait on forward and backward gait in Parkinson's disease. Gait Posture.

[19] Fasano A, Canning CG, Hausdorff JM, Lord S, Rochester L (2017). Falls in Parkinson's disease: A complex and evolving picture. Mov Disord.

[20] Brognara L, Navarro-Flores E, Iachemet L, Serra-Catalá N, Cauli O (2020). Beneficial Effect of Foot Plantar Stimulation in Gait Parameters in Individuals with Parkinson's Disease. Brain Sci.

[21] Petrucci MN, MacKinnon CD, Hsiao-Wecksler ET (2019). Modulation of anticipatory postural adjustments using a powered ankle orthosis in people with Parkinson's disease and freezing of gait. Gait Posture.

[22] Shah J, Pillai L, Williams DK (2018). Increased foot strike variability in Parkinson's disease patients with freezing of gait. Parkinsonism Relat Disord.

[23] Pouclet H, Derkinderen P, Lebouvier T (2013). Foot dystonia heralding levodopa induced dyskinesias in Parkinson disease. Clin Neurol Neurosurg.

[24] Mills R, Bahroo L, Pagan F (2015). An update on the use of botulinum toxin therapy in Parkinson's disease. Curr Neurol Neurosci Rep.

[25] Ashour R, Tintner R, Jankovic J (2005). Striatal deformities of the hand and foot in Parkinson's disease. Lancet Neurol.

[26] Gupta AD, Visvanathan R (2016). Botulinum toxin for foot dystonia in patients with Parkinson's disease having deep brain stimulation: A case series and a pilot study. J Rehabil Med.

[27] Spink MJ, Fotoohabadi MR, Wee E, Hill KD, Lord SR, Menz HB (2011). Foot and ankle strength, range of motion, posture, and deformity are associated with balance and functional ability in older adults. Arch Phys Med Rehabil.

[28] Chaiwanichsiri D, Janchai S, Tantisiriwat N (2009). Foot disorders and falls in older persons. Gerontology.

[29] Bowen C, Ashburn A, Cole M (2016). A survey exploring self-reported indoor and outdoor footwear habits, foot problems and fall status in people with stroke and Parkinson's. J Foot Ankle Res.

[30] Pereira MP, Orcioli-Silva D, de Sousa PN, Beretta VS, Gobbi LTB (2019). The effects of habitual footwear in gait outcomes in people with Parkinson's disease. Gait Posture.

[31] Kelsey JL, Procter-Gray E, Nguyen US, Li W, Kiel DP, Hannan MT (2010). Footwear and Falls in the Home Among Older Individuals in the MOBILIZE Boston Study. Footwear Sci.

[32] Barthel C, Nonnekes J, van Helvert M (2018). The laser shoes: A new ambulatory device to alleviate freezing of gait in Parkinson disease. Neurology.

[33] Pita Fernández, S. Epidemiología. Conceptos básicos. Madrid; DuPont Pharma.

[34] López López D, Grela Fariña M, Losa Iglesias ME, Calvo Lobo C, Rodríguez Sanz D, Palomo López P (2018). Clinical Aspects of Foot Health in Individuals with Alzheimer's Disease. Int J Environ Res Public Health.

[35] Tovaruela Carrión N, Bengoa Vallejo R, Losa Iglesias ME, Palomo López P, Munuera Martínez P, Pérez García S, López López D (2018). Accurately Determining Proper Shoe Size in Patients With Rheumatoid Arthritis. Rehabil Nurs.

[36] Centers of Disease Control. Body Mass Index: Consideration for Practitioners; Centers of Disease Control: Atlanta, GA,USA, 2011

[37] Calvo Lobo C, Ramos García A, Losa Iglesias ME, López López D, Rodríguez Sanz D, Romero Morales C (2018). The Relationship between Shoe Fitting and Foot Health of Persons with Down Syndrome: A Case Control Study. Int J Environ Res Public Health.

[38] Munuera Martínez P (2009). El primer radio. Biomecánica y ortopodología. 1 ed. Madrid: Santander EXA.

[39] Barouk P, Barouk LS (2014). Clinical diagnosis of gastrocnemius tightness. Foot Ankle Clin.

[40] Menz HB, Fotoohabadi MR, Wee E, Spink MJ (2010). Validity of self-assessment of hallux valgus using the Manchester scale. BMC Musculoskelet Disord.

[41] Menz HB, Munteanu SE (2005). Radiographic validation of the Manchester scale for the classification of hallux valgus deformity. Rheumatology.

[42] ACS I-S. Genuine Brannock Device. U.S.A. 2019.

[43] Harrison SJ, Cochrane L, Abboud RJ, Leese GP (2007). Do patients with diabetes wear shoes of the correct size?. Int J Clin Pract.

[44] Pita Fernández S (1996). Determinación del tamaño muestral. Cad Aten Primaria.

[45] Instituto Galego de Estadística. Santiago de Compostela: IGE; 2019.

[46] Instituto Nacional de Estadística. Encuesta de discapacidad, autonomía personal y situaciones de dependencia. Madrid: INE; 2008.

[47] Palomo López P, Bengoa Vallejo R, Losa Iglesias ME, Rodríguez Sanz D, Calvo Lobo C, López López D (2017). Footwear used by older people and a history of hyperkeratotic lesions on the foot: A prospective observational study. Medicine.

[48] Hatton AL, Rome K (2019). Falls, Footwear, and Podiatric Interventions in Older Adults. Clin Geriatr Med.

[49] Davis AM, Galna B, Murphy AT, Williams CM, Haines TP (2016). Effect of footwear on minimum foot clearance, heel slippage and spatiotemporal measures of gait in older women. Gait Posture.

[50] Buldt AK, Menz HB (2018). Incorrectly fitted footwear, foot pain and foot disorders: a systematic search and narrative review of the literature. J Foot Ankle Res.

[51] López López D, Losa Iglesias ME, Becerro de Bengoa Vallejo R (2015). Optimal choice of footwear in the elderly population. Geriatr Nurs.

[52] Harrison SJ, Cochrane L, Abboud RJ, Leese GP (2007). Do patients with diabetes wear shoes of the correct size?. Int J Clin Pract.

[53] Rodríguez-Sanz D, Tovaruela-Carrión N, López-López D, Palomo-López P (2017). Romero-Morales C, Navarro-Flores E, Calvo-Lobo C. Foot disorders in the elderly: A mini-review. Disease-a-Month.

[54] Navarro-Flores E, Romero-Morales C, Becerro de Bengoa-Vallejo R (2020). Sex Differences in Frail Older Adults with Foot Pain in a Spanish Population: An Observational Study. Int J Environ Res Public Health.

[55] Awale A, Hagedorn TJ, Dufour AB, Menz HB, Casey VA, Hannan MT (2017). Foot Function, Foot Pain, and Falls in Older Adults: The Framingham Foot Study. Gerontology.

